# *N*′-(5-Fluoro-2-oxo-2,3-dihydro-1*H*-indol-3-yl­idene)benzene­sulfono­hydrazide

**DOI:** 10.1107/S1600536808011136

**Published:** 2008-04-26

**Authors:** Hapipah M. Ali, Musalem Laila, Mohd. Razali Rizal, Seik Weng Ng

**Affiliations:** a Department of Chemistry, University of Malaya, 50603 Kuala Lumpur, Malaysia

## Abstract

The mol­ecule of the title compound, C_14_H_10_FN_3_O_3_S, consists of an indole unit and a phenylsulfonyl unit that are disposed in an approximately *trans* orientation relative to the N—N single bond. Two mol­ecules are arranged about a center of inversion, forming a hydrazide–carbonyl N—H⋯O hydrogen-bonded dimer; the dimers are linked by an indole–sulfonyl N—H⋯O hydrogen bond into a ribbon.

## Related literature

For the crystal structures of related 3-indole benzene­sulfonyl­hydrazones, see: Ali *et al.* (2007*a* ▶,*b* ▶,*c* ▶). For the crystal structure of 5-fluoro-1*H*-indole-2,3-dione, see: Naumov *et al.* (2000 ▶).
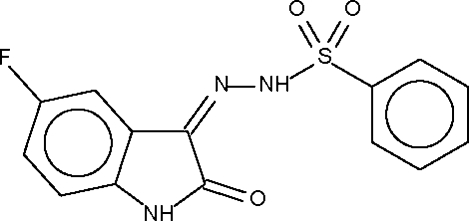


## Experimental

### 

#### Crystal data

C_14_H_10_FN_3_O_3_S
*M* *_r_* = 319.31
Monoclinic, 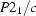

*a* = 8.2218 (2) Å
*b* = 16.4933 (3) Å
*c* = 10.8585 (2) Å
β = 110.249 (1)°
*V* = 1381.46 (5) Å^3^
*Z* = 4
Mo *K*α radiation
μ = 0.26 mm^−1^
*T* = 123 (2) K
0.50 × 0.20 × 0.15 mm

#### Data collection

Bruker SMART APEX diffractometer
Absorption correction: multi-scan (*SADABS*; Sheldrick, 1996 ▶) *T*_min_ = 0.816, *T*_max_ = 0.962
10513 measured reflections
3166 independent reflections
2741 reflections with *I* > 2σ(*I*)
*R*_int_ = 0.024

#### Refinement

*R*[*F* ^2^ > 2σ(*F* ^2^)] = 0.036
*wR*(*F* ^2^) = 0.145
*S* = 1.20
3166 reflections
207 parameters
2 restraints
H atoms treated by a mixture of independent and constrained refinement
Δρ_max_ = 0.54 e Å^−3^
Δρ_min_ = −0.59 e Å^−3^

### 

Data collection: *APEX2* (Bruker, 2005 ▶); cell refinement: *SAINT* (Bruker, 2005 ▶); data reduction: *SAINT*; program(s) used to solve structure: *SHELXS97* (Sheldrick, 2008 ▶); program(s) used to refine structure: *SHELXL97* (Sheldrick, 2008 ▶); molecular graphics: *X-SEED* (Barbour, 2001 ▶); software used to prepare material for publication: *publCIF* (Westrip, 2008 ▶).

## Supplementary Material

Crystal structure: contains datablocks global, I. DOI: 10.1107/S1600536808011136/sg2224sup1.cif

Structure factors: contains datablocks I. DOI: 10.1107/S1600536808011136/sg2224Isup2.hkl

Additional supplementary materials:  crystallographic information; 3D view; checkCIF report

## Figures and Tables

**Table 1:** Hydrogen-bond geometry (Å, °)

*D*—H⋯*A*	*D*—H	H⋯*A*	*D*⋯*A*	*D*—H⋯*A*
N1—H1n⋯O3^i^	0.88 (1)	2.10 (2)	2.896 (2)	151 (2)
N3—H3n⋯O1^ii^	0.88 (1)	2.22 (2)	2.986 (2)	145 (2)

Symmetry codes: (i) 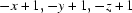; (ii) 
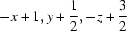
.

**Fig. 1.**
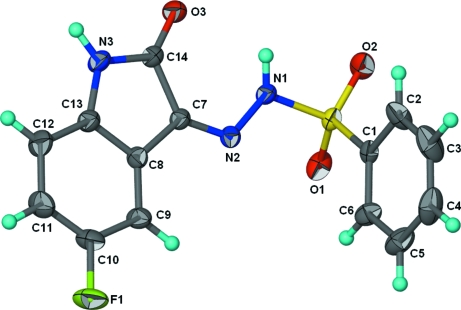
Thermal ellipsoid plot of C14H10FN3O3S. Displacement ellipsoids are drawn at the 70% probability level, and H atoms are shown as spheres of arbitrary radii.

**Fig. 2.**
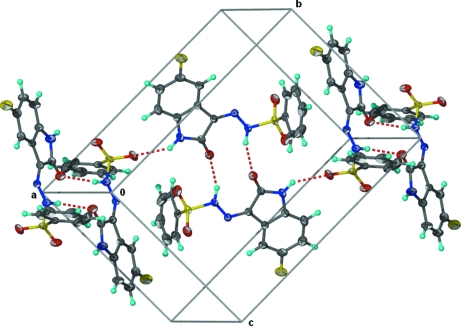
Ribbon structure of C10H10FN3O3S.

## supplementary crystallographic information

### Comment

We have reported the crystal structures of 3-indole benzenesulfonohydrazides (Ali *et al.*, 2007*a*, 2007*b*, 2007*c*). The studies continue with the benzenesulfonohydrazide that is obtained by condensing benzenesulfonohydrazine with a substituted 1*H*-indol-2,3-dione, 5-fluroisatin. This compound exists as a hydrogen-bonded dimer (Naumov *et al.*, 2000). The title compound (Scheme I) has the indolyl fused-ring portion and the phenylsulfonyl portion disposed in an approximately *trans*-alignment relative to the N–N single-bond (Fig. 1). Two molecules are arranged about a center-of-inversion to form an *N*–H_hydrazide_···O_carbonyl_ hydrogen-bonded dimer; the dimers are linked by another *N*–H_indole_···O_sulfonyl_ hydrogen bond into a ribbon structure (Fig. 2).

### Experimental

Benzenesulfonyl hydrazide (0. 69 g, 4 mmol) and 5-fluoroisatin (0.66 g, 4 mmol) were heated in ethanol (50 ml) for an hour. The solution when cooled afforded yellow crystals.

### Refinement

The carbon-bound H atoms were placed at calculated positions (C–H 0.95 Å), and were included in the refinement in the riding model approximation with *U*(H) set to 1.2*U*_eq_(C). The amino H atoms were located in a difference Fouier map, and were refined with a distance restraint of N–H 0.88±0.01 Å.

### Crystal data

**Table:**

C_14_H_10_FN_3_O_3_S	*F*_000_ = 656
*M**_r_* = 319.31	*D*_x_ = 1.535 Mg m^−^^3^
Monoclinic, *P*2_1_/*c*	Mo *K*α radiation λ = 0.71073 Å
Hall symbol: -P 2ybc	Cell parameters from 6063 reflections
*a* = 8.2218 (2) Å	θ = 3.0–31.3º
*b* = 16.4933 (3) Å	µ = 0.26 mm^−^^1^
*c* = 10.8585 (2) Å	*T* = 123 (2) K
β = 110.249 (1)º	Irregular block, yellow
*V* = 1381.46 (5) Å^3^	0.50 × 0.20 × 0.15 mm
*Z* = 4	

### Data collection

**Table:**

Bruker SMART APEX diffractometer	3166 independent reflections
Radiation source: medium-focus sealed tube	2741 reflections with *I* > 2σ(*I*)
Monochromator: graphite	*R*_int_ = 0.024
*T* = 123(2) K	θ_max_ = 27.5º
φ and ω scans	θ_min_ = 2.4º
Absorption correction: multi-scan(SADABS; Sheldrick, 1996)	*h* = −10→10
*T*_min_ = 0.816, *T*_max_ = 0.962	*k* = −21→21
10513 measured reflections	*l* = −13→14

### Refinement

**Table:**

Refinement on *F*^2^	Secondary atom site location: difference Fourier map
Least-squares matrix: full	Hydrogen site location: inferred from neighbouring sites
*R*[*F*^2^ > 2σ(*F*^2^)] = 0.036	H atoms treated by a mixture of independent and constrained refinement
*wR*(*F*^2^) = 0.145	*w* = 1/[σ^2^(*F*_o_^2^) + (0.0922*P*)^2^ + 0.2432*P*] where *P* = (*F*_o_^2^ + 2*F*_c_^2^)/3
*S* = 1.20	(Δ/σ)_max_ = 0.001
3166 reflections	Δρ_max_ = 0.54 e Å^−^^3^
207 parameters	Δρ_min_ = −0.59 e Å^−^^3^
2 restraints	Extinction correction: none
Primary atom site location: structure-invariant direct methods	

### Special details

**Table:**

Experimental. A medium-focus collimator of 0.8 mm diameter was used on the diffractometer to measure the somewhat large crystal.
Geometry. All e.s.d.'s (except the e.s.d. in the dihedral angle between two l.s. planes) are estimated using the full covariance matrix. The cell e.s.d.'s are taken into account individually in the estimation of e.s.d.'s in distances, angles and torsion angles; correlations between e.s.d.'s in cell parameters are only used when they are defined by crystal symmetry. An approximate (isotropic) treatment of cell e.s.d.'s is used for estimating e.s.d.'s involving l.s. planes.

### Fractional atomic coordinates and isotropic or equivalent isotropic displacement parameters (Å^2^)

**Table:**

	*x*	*y*	*z*	*U*_iso_*/*U*_eq_	
S1	0.65386 (5)	0.28692 (2)	0.54366 (4)	0.01609 (16)	
O1	0.61921 (18)	0.21966 (7)	0.61409 (14)	0.0248 (3)	
O2	0.56524 (17)	0.29375 (8)	0.40497 (13)	0.0255 (3)	
O3	0.42474 (17)	0.51614 (8)	0.60498 (13)	0.0240 (3)	
N1	0.59808 (19)	0.37034 (9)	0.60384 (14)	0.0183 (3)	
H1N	0.557 (3)	0.4089 (11)	0.546 (2)	0.036 (7)*	
N2	0.67998 (18)	0.38149 (8)	0.73560 (14)	0.0172 (3)	
N3	0.5337 (2)	0.57008 (9)	0.81558 (16)	0.0232 (3)	
H3N	0.478 (3)	0.6168 (9)	0.799 (2)	0.038 (7)*	
C1	0.8794 (2)	0.29132 (9)	0.58025 (17)	0.0165 (3)	
C2	0.9467 (3)	0.33843 (13)	0.50285 (19)	0.0277 (4)	
H2	0.8730	0.3696	0.4318	0.033*	
C3	1.1256 (3)	0.33841 (16)	0.5329 (2)	0.0368 (5)	
H3	1.1754	0.3703	0.4823	0.044*	
C4	1.2315 (3)	0.29211 (13)	0.6361 (2)	0.0349 (5)	
H4	1.3530	0.2914	0.6541	0.042*	
C5	1.1624 (2)	0.24721 (12)	0.7127 (2)	0.0300 (4)	
H5	1.2364	0.2164	0.7841	0.036*	
C6	0.9846 (2)	0.24692 (10)	0.68578 (19)	0.0227 (4)	
H6	0.9360	0.2167	0.7390	0.027*	
C7	0.6472 (2)	0.44753 (10)	0.78623 (17)	0.0174 (3)	
C8	0.7275 (2)	0.47125 (10)	0.92317 (17)	0.0182 (4)	
C9	0.8561 (2)	0.43576 (10)	1.02774 (17)	0.0219 (4)	
H9	0.9069	0.3853	1.0192	0.026*	
C10	0.9065 (3)	0.47769 (11)	1.14518 (18)	0.0252 (4)	
C11	0.8372 (3)	0.55171 (11)	1.16168 (19)	0.0285 (4)	
H11	0.8765	0.5779	1.2447	0.034*	
C12	0.7090 (3)	0.58763 (11)	1.05538 (19)	0.0276 (4)	
H12	0.6597	0.6385	1.0641	0.033*	
C13	0.6564 (2)	0.54660 (10)	0.93715 (18)	0.0204 (4)	
C14	0.5206 (2)	0.51454 (10)	0.72023 (17)	0.0187 (4)	
F1	1.03509 (17)	0.44573 (7)	1.25055 (11)	0.0374 (3)	

### Atomic displacement parameters (Å^2^)

**Table:**

	*U*^11^	*U*^22^	*U*^33^	*U*^12^	*U*^13^	*U*^23^
S1	0.0133 (2)	0.0151 (2)	0.0192 (3)	−0.00149 (13)	0.00483 (17)	−0.00402 (14)
O1	0.0232 (7)	0.0164 (6)	0.0388 (8)	−0.0042 (5)	0.0157 (6)	−0.0018 (5)
O2	0.0199 (7)	0.0309 (7)	0.0207 (7)	−0.0001 (5)	0.0006 (5)	−0.0086 (5)
O3	0.0257 (7)	0.0238 (6)	0.0195 (6)	0.0050 (5)	0.0039 (5)	−0.0003 (5)
N1	0.0202 (7)	0.0158 (7)	0.0180 (7)	0.0032 (5)	0.0053 (6)	−0.0009 (5)
N2	0.0179 (7)	0.0163 (7)	0.0179 (7)	−0.0007 (5)	0.0069 (6)	−0.0010 (5)
N3	0.0250 (8)	0.0194 (7)	0.0219 (8)	0.0074 (6)	0.0039 (6)	−0.0024 (6)
C1	0.0132 (8)	0.0191 (8)	0.0172 (8)	−0.0017 (6)	0.0053 (6)	−0.0053 (6)
C2	0.0251 (9)	0.0409 (11)	0.0185 (9)	−0.0057 (8)	0.0093 (7)	0.0000 (8)
C3	0.0284 (10)	0.0596 (14)	0.0287 (11)	−0.0140 (10)	0.0178 (9)	−0.0058 (10)
C4	0.0153 (9)	0.0477 (13)	0.0424 (12)	−0.0030 (8)	0.0108 (9)	−0.0180 (10)
C5	0.0196 (9)	0.0262 (10)	0.0370 (11)	0.0035 (7)	0.0005 (8)	−0.0062 (8)
C6	0.0202 (8)	0.0180 (8)	0.0269 (9)	0.0000 (6)	0.0042 (7)	0.0001 (7)
C7	0.0182 (8)	0.0154 (7)	0.0191 (8)	0.0007 (6)	0.0072 (7)	0.0000 (6)
C8	0.0223 (8)	0.0144 (7)	0.0183 (8)	−0.0007 (6)	0.0076 (7)	−0.0013 (6)
C9	0.0266 (9)	0.0166 (8)	0.0207 (9)	0.0012 (6)	0.0059 (7)	0.0019 (6)
C10	0.0287 (9)	0.0225 (9)	0.0197 (9)	−0.0010 (7)	0.0023 (7)	0.0037 (7)
C11	0.0364 (11)	0.0251 (9)	0.0202 (9)	−0.0022 (8)	0.0052 (8)	−0.0058 (7)
C12	0.0327 (10)	0.0217 (9)	0.0251 (10)	0.0039 (8)	0.0059 (8)	−0.0073 (7)
C13	0.0216 (8)	0.0180 (8)	0.0206 (9)	0.0019 (6)	0.0060 (7)	−0.0008 (6)
C14	0.0184 (8)	0.0172 (8)	0.0206 (9)	0.0016 (6)	0.0069 (7)	0.0005 (6)
F1	0.0471 (8)	0.0298 (6)	0.0211 (6)	0.0055 (5)	−0.0061 (5)	0.0026 (5)

### Geometric parameters (Å, °)

**Table:**

S1—O1	1.4309 (13)	C4—C5	1.375 (3)
S1—O2	1.4325 (14)	C4—H4	0.9500
S1—N1	1.6545 (14)	C5—C6	1.388 (3)
S1—C1	1.7582 (17)	C5—H5	0.9500
O3—C14	1.227 (2)	C6—H6	0.9500
N1—N2	1.367 (2)	C7—C8	1.456 (2)
N1—H1N	0.876 (10)	C7—C14	1.518 (2)
N2—C7	1.290 (2)	C8—C9	1.385 (2)
N3—C14	1.358 (2)	C8—C13	1.404 (2)
N3—C13	1.410 (2)	C9—C10	1.382 (3)
N3—H3N	0.884 (10)	C9—H9	0.9500
C1—C6	1.383 (2)	C10—F1	1.366 (2)
C1—C2	1.392 (3)	C10—C11	1.385 (3)
C2—C3	1.392 (3)	C11—C12	1.397 (3)
C2—H2	0.9500	C11—H11	0.9500
C3—C4	1.386 (3)	C12—C13	1.382 (3)
C3—H3	0.9500	C12—H12	0.9500
			
O1—S1—O2	119.99 (8)	C1—C6—C5	118.97 (18)
O1—S1—N1	107.46 (8)	C1—C6—H6	120.5
O2—S1—N1	103.90 (8)	C5—C6—H6	120.5
O1—S1—C1	107.50 (8)	N2—C7—C8	125.06 (15)
O2—S1—C1	110.32 (8)	N2—C7—C14	128.61 (16)
N1—S1—C1	106.93 (7)	C8—C7—C14	106.32 (14)
N2—N1—S1	114.96 (11)	C9—C8—C13	120.91 (16)
N2—N1—H1N	125.5 (17)	C9—C8—C7	132.16 (16)
S1—N1—H1N	114.2 (17)	C13—C8—C7	106.84 (15)
C7—N2—N1	117.39 (14)	C10—C9—C8	116.43 (16)
C14—N3—C13	111.76 (15)	C10—C9—H9	121.8
C14—N3—H3N	122.4 (17)	C8—C9—H9	121.8
C13—N3—H3N	125.6 (17)	F1—C10—C9	118.54 (17)
C6—C1—C2	122.02 (17)	F1—C10—C11	117.74 (17)
C6—C1—S1	118.29 (14)	C9—C10—C11	123.70 (17)
C2—C1—S1	119.69 (14)	C10—C11—C12	119.57 (17)
C1—C2—C3	117.85 (19)	C10—C11—H11	120.2
C1—C2—H2	121.1	C12—C11—H11	120.2
C3—C2—H2	121.1	C13—C12—C11	117.64 (17)
C4—C3—C2	120.4 (2)	C13—C12—H12	121.2
C4—C3—H3	119.8	C11—C12—H12	121.2
C2—C3—H3	119.8	C12—C13—C8	121.74 (17)
C5—C4—C3	120.72 (19)	C12—C13—N3	128.89 (16)
C5—C4—H4	119.6	C8—C13—N3	109.37 (15)
C3—C4—H4	119.6	O3—C14—N3	128.06 (16)
C4—C5—C6	119.97 (19)	O3—C14—C7	126.22 (15)
C4—C5—H5	120.0	N3—C14—C7	105.70 (15)
C6—C5—H5	120.0		
			
O1—S1—N1—N2	57.51 (14)	C14—C7—C8—C13	−0.74 (19)
O2—S1—N1—N2	−174.35 (12)	C13—C8—C9—C10	1.2 (3)
C1—S1—N1—N2	−57.66 (14)	C7—C8—C9—C10	177.29 (18)
S1—N1—N2—C7	176.78 (12)	C8—C9—C10—F1	−178.89 (16)
O1—S1—C1—C6	−14.56 (16)	C8—C9—C10—C11	−0.6 (3)
O2—S1—C1—C6	−147.07 (13)	F1—C10—C11—C12	178.16 (18)
N1—S1—C1—C6	100.57 (14)	C9—C10—C11—C12	−0.1 (3)
O1—S1—C1—C2	165.08 (14)	C10—C11—C12—C13	0.3 (3)
O2—S1—C1—C2	32.58 (16)	C11—C12—C13—C8	0.3 (3)
N1—S1—C1—C2	−79.78 (16)	C11—C12—C13—N3	−178.45 (19)
C6—C1—C2—C3	1.4 (3)	C9—C8—C13—C12	−1.1 (3)
S1—C1—C2—C3	−178.20 (16)	C7—C8—C13—C12	−178.09 (17)
C1—C2—C3—C4	0.5 (3)	C9—C8—C13—N3	177.87 (16)
C2—C3—C4—C5	−1.8 (3)	C7—C8—C13—N3	0.9 (2)
C3—C4—C5—C6	1.0 (3)	C14—N3—C13—C12	178.14 (19)
C2—C1—C6—C5	−2.2 (3)	C14—N3—C13—C8	−0.8 (2)
S1—C1—C6—C5	177.47 (14)	C13—N3—C14—O3	178.88 (18)
C4—C5—C6—C1	0.9 (3)	C13—N3—C14—C7	0.3 (2)
N1—N2—C7—C8	−177.31 (15)	N2—C7—C14—O3	0.9 (3)
N1—N2—C7—C14	3.5 (3)	C8—C7—C14—O3	−178.35 (17)
N2—C7—C8—C9	3.5 (3)	N2—C7—C14—N3	179.59 (17)
C14—C7—C8—C9	−177.21 (18)	C8—C7—C14—N3	0.29 (19)
N2—C7—C8—C13	179.93 (17)		

### Hydrogen-bond geometry (Å, °)

**Table:**

*D*—H···*A*	*D*—H	H···*A*	*D*···*A*	*D*—H···*A*
N1—H1n···O3^i^	0.88 (1)	2.10 (2)	2.896 (2)	151 (2)
N3—H3n···O1^ii^	0.88 (1)	2.22 (2)	2.986 (2)	145 (2)

Symmetry codes: (i) −*x*+1, −*y*+1, −*z*+1; (ii) −*x*+1, *y*+1/2, −*z*+3/2.

## References

[bb1] Ali, H. M., Nazzatush Shimar, J., Wan Jefrey, B. & Ng, S. W. (2007*a*). *Acta Cryst.* E**63**, o1807–o1808.

[bb2] Ali, H. M., Yusnita, J., Wan Jefrey, B. & Ng, S. W. (2007*b*). *Acta Cryst.* E**63**, o1621–o1622.

[bb3] Ali, H. M., Yusnita, J., Wan Jefrey, B. & Ng, S. W. (2007*c*). *Acta Cryst.* E**63**, o3513.

[bb4] Barbour, L. J. (2001). *J. Supramol. Chem.* **1**, 189–191.

[bb5] Bruker (2005). *APEX2* and *SAINT* Bruker AXS Inc., Madison, Wisconsin, USA.

[bb6] Naumov, P., Anastasova, F., Drew, M. G. B. & Ng, S. W. (2000). *Acta Cryst.* C**56**, e406–e407.

[bb7] Sheldrick, G. M. (1996). *SADABS* University of Göttingen, Germany.

[bb8] Sheldrick, G. M. (2008). *Acta Cryst.* A**64**, 112–122. 10.1107/S0108767307043930 18156677

[bb9] Westrip, S. P. (2008). *publCIF* In preparation.

